# Low-voltage high-performance flexible digital and analog circuits based on ultrahigh-purity semiconducting carbon nanotubes

**DOI:** 10.1038/s41467-019-10145-9

**Published:** 2019-05-14

**Authors:** Ting Lei, Lei-Lai Shao, Yu-Qing Zheng, Gregory Pitner, Guanhua Fang, Chenxin Zhu, Sicheng Li, Ray Beausoleil, H.-S. Philip Wong, Tsung-Ching Huang, Kwang-Ting Cheng, Zhenan Bao

**Affiliations:** 1 Department of Chemical Engineering, Stanford University, Stanford, CA 94305 USA; 2 Department of Materials Science and Engineering, College of Engineering, Peking University, 100871 Beijing, China; 3 Department of Electrical and Computer Engineering, University of California, Santa Barbara, CA 93106 USA; 4 Hewlett Packard Labs, Palo Alto, CA 94304 USA; 5 Department of Electrical Engineering, Stanford University, Stanford, CA 94305 USA; 6 Department of Materials Science & Engineering, Stanford University, Stanford, CA 94305 USA; 7 School of Engineering, Hong Kong University of Science and Technology, Hong Kong, 999077 China

**Keywords:** Electrical and electronic engineering, Materials for devices

## Abstract

Carbon nanotube (CNT) thin-film transistor (TFT) is a promising candidate for flexible and wearable electronics. However, it usually suffers from low semiconducting tube purity, low device yield, and the mismatch between p- and n-type TFTs. Here, we report low-voltage and high-performance digital and analog CNT TFT circuits based on high-yield (19.9%) and ultrahigh purity (99.997%) polymer-sorted semiconducting CNTs. Using high-uniformity deposition and pseudo-CMOS design, we demonstrated CNT TFTs with good uniformity and high performance at low operation voltage of 3 V. We tested forty-four 2-µm channel 5-stage ring oscillators on the same flexible substrate (1,056 TFTs). All worked as expected with gate delays of 42.7 ± 13.1 ns. With these high-performance TFTs, we demonstrated 8-stage shift registers running at 50 kHz and the first tunable-gain amplifier with 1,000 gain at 20 kHz. These results show great potentials of using solution-processed CNT TFTs for large-scale flexible electronics.

## Abstract (web summary)

Carbon nanotube thin-film transistor is promising for solution-processed, large-scale flexible electronics, but the device yields remain poor to date. Lei et al. show low-voltage flexible digital and analog circuits based on high-purity and high-yield separation of semiconducting carbon nanotubes.

## Introduction

High-performance flexible electronics are highly desirable for applications in wearables, healthcare, prosthetics, and robotics^1–4^. Carbon nanotube (CNT) thin-film transistor (TFT) is a promising candidate for high-performance flexible electronics because of its high carrier mobility, high mechanical flexibility/stretchability, and compatibility with low cost printing processes^5–9^. CNT TFT-based circuits, such as flexible logic circuits^7,10^, and systems, such as flexible and stretchable sensors^11^ and bio-medical devices^12^, have been previously reported. However, to date, CNT TFT circuits were only implemented as simple ring oscillators running at low frequencies (<100 kHz)^10^ or small-scale logic circuits with a limited number of transistors (<50 transistors)^7^. The major obstacles include process complexity and low circuit yield due to the hybrid-integration of different types of TFT devices (e.g. p-type CNT and n-type IGZO), as well as using slower and less reliable n-type CNT TFTs to realize the complementary logic. To enable large-scale flexible systems with low manufacturing costs, sensors, amplifiers, and sensor interface circuits need to be integrated and fabricated on the same substrate with hundreds to thousands of transistors^13^. Flexible amplifiers and sensor interface circuits, such as drivers and multiplexers are also essential for flexible/silicon hybrid systems^14^. However, a demonstrator of CNT TFT circuits with medium-to-large scale high-performance digital and analog circuits for sensor interface and internet-of-things (IoT) applications is still missing^7,15^.

Even though much progresses have been made in CNT growth and sorting^16–18^, the typical semiconducting purity of <99.9% of the solution-purified CNTs is still insufficient for large-scale circuits. Furthermore, circuit operating speed is dependent on transistor channel length. Therefore, lack of high-purity and high-uniformity CNT network films are the current main obstacles for large-scale CNT flexible circuits with required speeds. In addition, in order to realize complementary metal oxide semiconductor (CMOS)-like CNT circuits^19,20^, unstable n-type dopants and low-work-function metals are often used, which inevitably increases the fabrication complexity and negatively impacts the device yield and uniformity. We have made many efforts on using n-type dopant for CMOS circuit fabrication. Although small-scale logics were successfully achieved in our previous work^20^, large-scale integration turned out to be very challenging due to the large device-to-device variations (up to 50% for mobility) of n-type TFTs. Our simulation results suggest that large CNT TFT device variations (e.g. *δ* > 20%*μ*) could result in a drop of noise margins ~50% and an increasing circuit failure rate up to 15/500 (Supplementary Note 1). Therefore, large-scale circuits require more emphasis on uniformity and reproducibility of device performance.

Here we report a polymer-sorting technique that achieves an ultra-high selectivity of 99.997% and a high sorting yield of 19.9%, much higher than previous reported methods (usually selectivity < 99.9% and yield < 5%)^21–23^. We increase the uniformity and long-time bias stability of the flexible circuits by introducing a new type of self-assembly monolayer (SAM) for CNT absorption, adding additional barrier layers, and using bottom-contact device configuration. With the pseudo-CMOS design style^24,25^, we demonstrate high-performance five-stage pseudo-CMOS-based ring oscillators running up to 3.5 MHz, an eight-stage shift register consisting of 304 CNT TFTs operating at 50 kHz, as well as a tunable-gain amplifier with 1000 voltage gain at 20 kHz^26^. These circuits are foundational building blocks for implementing flexible sensor arrays and flexible displays (Supplementary Fig. 1).

## Results

### Improve the sorting selectivity of CNTs

**Fig. 1:**
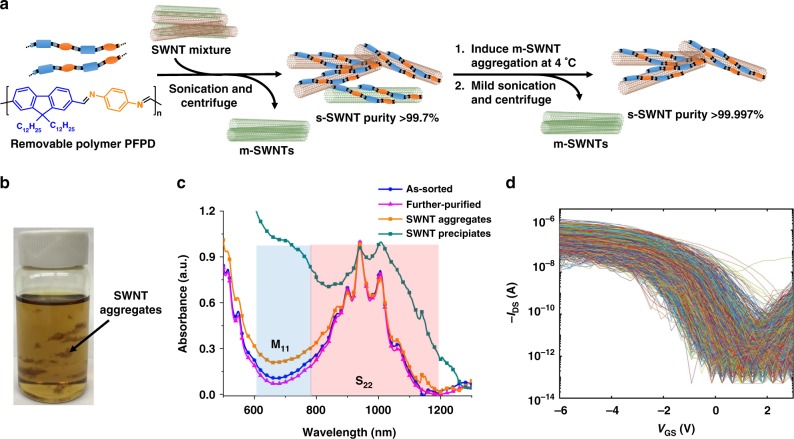
CNT purity improvement by inducing further aggregation of the metallic CNTs. **a** Schematic presentation of the sorting process modifying our previously reported method^26^. **b** A photograph showing some CNT aggregates formed after the solution being stored at 4 °C for 24 h. **c** Absorption spectra of as-sorted SWNTs (blue), further purified SWNTs (magenta), isolated SWNT aggregates (orange), and SWNT precipitates after bath sonication of the solution with aggregates followed by centrifugation (turquoise). M_11_ is the first inter-band transition of m-SWNTs. S_22_ is the second inter-band transition of s-SWNTs. **d** Electrical measurement of the final purified SWNTs (device batch #2). Two batches of short-channel (500 nm) transistors were fabricated (see “Methods” and Supplementary Fig. 4 for details). About 34,590 CNTs were tested, we found only 1 metallic tube. The purity is thus estimated to be 99.997%

Recent studies have shown that the selectivity of semiconducting single-walled CNTs (s-SWNTs) can be as high as 99.9%^9,19,21,27^. However, even higher s-SWNT purity is needed in order to realize large-scale circuits containing hundreds to thousands of CNT TFTs. This is especially important for short-channel-length high-speed circuits, because m-SWNTs are more likely to bridge the source–drain electrodes in short-channel CNT TFTs. Compared with other CNT sorting methods, polymer wrapping method provides many advantages, such as high selectivity (>99%), high CNT concentration (~0.1 mg/mL), and short processing time (<1 h)^16–18^. We found that the sorting mechanism of polymer wrapping might be due to the different aggregation behaviors between s-SWNT/polymer complex and m-SWNT/polymer complex^28^. Theoretical studies also suggest that m-SWNT/polymer complex have stronger charge transfer interactions than its s-SWNT counterpart^29^. Therefore, the more polar m-SWNT/polymer complex tends to aggregate and form sediments during centrifuge, leaving s-SWNT/polymer complex in supernatant. Based on these studies, we tried to further improve the s-SWNT purity by inducing the metallic tube aggregation. We used a removable and recyclable imine polymer (poly[(9,9-di-n-dodecyl-2,7-fluorendiyl-dimethine)-(1,4-phenylene-dinitrilomethine)], PFPD, Fig. 1a) for selectively dispersing s-SWNTs^26^. The polymer exhibited a high selectivity of 99.7% and high yield of 23.7 wt% reported previously for initial s-SWNT sorting^26^. To further improve the purity, the solution was stored in a fridge (4 °C) for 24 h and visible aggregates were observed (Fig. 1b). After centrifugation, the aggregates can be removed. UV–vis absorption spectroscopy showed stronger absorption in the 600–750 nm range for the aggregates (orange line in Fig. 1c). This range is attributed to the first inter-band transition of m-SWNTs (M_11_), suggesting that the aggregates contain a higher metallic content. After mildly sonicating the solution in a bath sonicator, the aggregates disappeared. After centrifugation, we obtained some precipitates that have stronger absorption in the M_11_ region, suggesting even larger amount of m-SWNTs (turquoise line in Fig. 1c). In contrast, the supernatant showed lower absorption intensity in M_11_ region (magenta line in Fig. 1c), suggesting higher selectivity compared with the original solution. These results indicate that after storing the initial sorted solution at a lower temperature, both m-SWNTs and s-SWNTs aggregated in the solution. After a mild sonication, s-SWNTs can be re-dispersed whereas m-SWNTs remain aggregated and can be centrifuged away. On the other hand, direct centrifugation without mild sonication results in significant loss of s-SWNTs. Using mild sonication and centrifugation, only 16% of the s-SWNTs were lost during the additional purification step (Supplementary Fig. 2). Thus, the yield of the s-SWNT after twice purification remains very high and can be estimated at about 19.9%. We also tried the multiple centrifugation method to improve the purity, however, although multiple centrifugations can lead to a higher purity, the total yield (<5%) is significantly lower than our method.

Since it is not possible to obtain an exact purity value using spectroscopic method for s-SWNTs with > 99% purity due to the complicated nature of SWNT absorption spectrum, direct electrical measurement of single tubes using short channels (<500 nm, shorter than the length of CNTs) has been used for quantitative purity analysis^27^. In our evaluation, two batches of short-channel transistors were fabricated (Supplementary Fig. 3). 1023 FETs in batch #1 (density: 4.5 CNTs/FET), and 5170 FETs in batch #2 (density: 5.8 CNTs/FET) were used for evaluation (Fig. 1d and Supplementary Fig. 4, see “Methods” for details). Scanning electron microscope (SEM) was used to determine average tube density for each batch (Supplementary Fig. 3). Thus, totally ~34,590 SWNTs were tested. Among these tubes, we found only one shorted device defined as with an on/off ratio < 100 (Supplementary Fig. 4). Since our s-SWNT purity is very high, we assume that any shorted device was caused by one m-SWNT. Thus, the s-SWNT purity is estimated to be (1–1/34,590) × 100% = 99.997%. To the best of our knowledge, this is the highest s-SWNT purity ever reported^7,22^. We would like to note that high-yield separation of s-SWNTs is very important for low-cost and large-area flexible electronics, and this secondary purification method is not limited to our polymer system and can be applied to other polymer sorting methods.

### Device fabrication and stability consideration

**Fig. 2:**
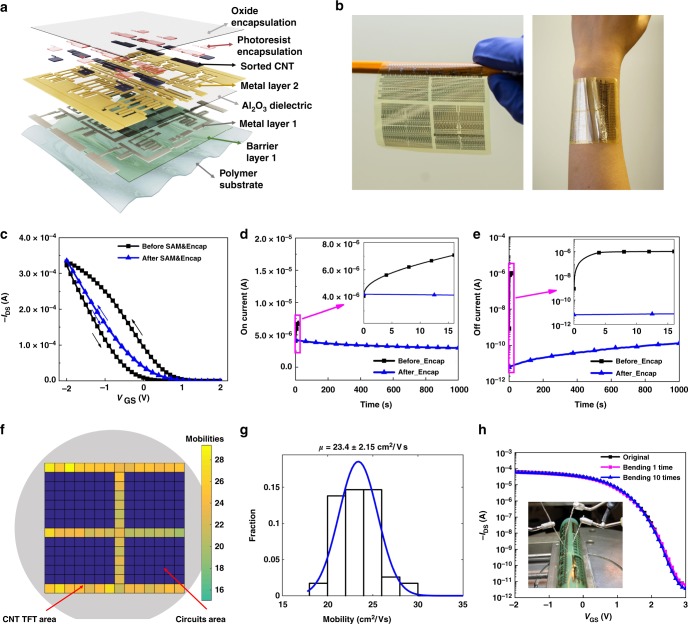
Device layer structures and electrical characterizations of CNT TFTs on a plastic substrate. **a** Layer structures of the CNT TFT device. **b** Photos of CNT TFT devices fabricated on a 10-μm flexible polyimide substrate. The thin flexible devices show good conformal attachment to human skin. **c** Transfer characterizations of CNT TFTs before and after SAM layer modification and Al_2_O_3_ encapsulation (*V*_DS_ = −1 V). **d** On-current bias stability of a CNT TFT before and after Al_2_O_3_ encapsulation. Inset shows the enlarged area (0–16 s) of the bias stability test. **e** Off-current bias stability of a CNT TFT before and after Al_2_O_3_ encapsulation. Inset shows the enlarged area (0–16 s). **f**, **g** Mobility statistics from 56 CNT TFTs tested on a 4-inch wafer. The 56 CNT TFTs are distributed on the yellow areas. The colored scale bar indicates the mobility values. Dark blue areas are circuit areas and were not tested. The average mobility of CNT TFTs is 23.4 cm^2^ V^−1^ s^−1^ with a standard deviation of 2.15 cm^2^ V^-1^ s^-1^. **h** Bending test of a flexible TFT with a bending radius of 5 mm. Only slight current changes (<10%) were observed after 10 times of repeated bending. Inset shows the bending and testing setup image

s-SWNT TFTs were fabricated on a 4-inch carrier wafer using the device structure shown in Fig. 2a. A flexible substrate, either a 10 µm polyimide or a 1 µm parylene, was deposited on a carrier wafer. A thin SiN_*x*_ layer (50 nm) was subsequently deposited to prevent moisture diffusion through the polymer substrate. After lithographic patterning and deposition of the gate electrodes (Cr 35 nm, Metal Layer 1), 25 nm Al_2_O_3_ and 3 nm SiO_2_ were deposited as the dielectric layer using atomic layer deposition (ALD) and patterned with photolithography and wet etching. After Pd drain/source contact electrode patterning and deposition (Metal Layer 2), the SiO_2_ layer was functionalized with 11-(2-methoxyethoxy) undecyltrimethoxysilane to form a SAM (Supplementary Fig. 5). Then, the high-purity CNTs were deposited by soaking the wafer in the purified CNT solution, followed by removing the polymer residues and thermal annealing to increase the CNT adhesion (detailed procedures are provided in “Methods”). We found that repeating the above deposition once can increase the uniformity of the CNT networks and thus the device performance uniformity. The deposited CNTs have diameters of 1.3–1.5 nm^26^ and lengths of 0.5–2 μm, and the CNT line density is about 35–40 CNTs/μm (Supplementary Fig. 6). Note that the wrapping polymers were almost completely removed after acid rinsing (Supplementary Fig. 7), which can further improve the CNT device performance and device uniformity. A negative photoresist was spin-coated on top of the CNT layer for both CNT patterning and encapsulation. Finally, a 40-nm Al_2_O_3_ encapsulation layer was coated by ALD to encapsulate all the devices. The flexible devices are subsequently characterized before and after peeling off (Fig. 2b).

The SAM molecule design is novel. It has a hydrophobic base to reduce electrical hysteresis and a hydrophilic tail to help CNT absorption. It has been shown previously that hydrophobic surfaces help reduce interfacial traps on the dielectric surface^30^. The methyl ethylene glycol hydrophilic part is designed to enhance the CNT absorption, because we found that pure hydrophobic SAMs significantly decrease the CNT density. By combining the SAM modification with polymer/Al_2_O_3_ encapsulation, the electrical hysteresis can be significantly reduced (Fig. 2c). In addition, the encapsulation was found necessary to provide a much better bias stability of the CNT TFTs (Fig. 2d, e). Before encapsulation, the CNT transistors showed very large on-current and off-current increase within 15 s of biasing the devices. In contrast, after encapsulation, the device became much more stable over time upon gate and drain biasing. Only slightly on-current decrease and off-current increase were observed. We found that relying only on polymer encapsulation is not sufficient because moisture and oxygen can still diffuse through. Similarly, we also found that it was necessary to include a barrier layer above the plastic substrate to ensure low hysteresis and stable operation. These results indicate that the bias instability is largely due to oxygen and water doping, and proper encapsulation is important for practical applications of CNT TFTs. For the device structure, we chose the bottom-contact configuration, where source/drain electrodes are deposited below CNT networks as shown in Fig. 2a. Because top-contact configuration needs photolithography on CNTs, which will cause significant CNT loss during the lift-off process, and finally resulting in lower device uniformity and yield. Although bottom-contact configuration tends to show slightly lower performance than top-contact due to larger contact resistance, however, after systematic optimization and using pseudo-CMOS design, we achieved comparable or even better performance than other top-contact and CMOS-design circuits^7,10^ (see the following circuits section). We employ a photoresist layer for CNT TFT encapsulation. Since the photoresist contains a small amount of acid, the CNT TFT are slightly p-doped. However, decreasing the acid amount or using other polymer encapsulation layers can control the threshold voltages (*V*_Th_). Similarly, using base or n-type dopant could also control the *V*_Th_. Thus, the *V*_Th_ of our CNT TFTs can be controlled by different chemical dopants or different types of polymer encapsulation layers.

To obtain the electrical performance statistics, over 50 transistors were measured across a 4-inch wafer (Fig. 2f and Supplementary Figs. 8–11). We found that our fabrication method provided very high uniformity cross the 4-inch wafer with an average mobility of 23.4 cm^2^ V^−1^ s^−1^, high on/off ratios of > 10^5^ and a small standard deviation of <10% (Fig. 2g). To the best of our knowledge, these values are among the highest performance and the best uniformity for wafer-scale-fabricated flexible CNT TFTs (Supplementary Tables 1 and 2)^10^. The performance and uniformity of our CNT TFTs are already comparable and even better than some oxide-based TFTs used for display applications^31^. Note that higher mobilities up to 49 cm^2^ V^−1^ s^−1^ has been achieved by increasing the CNT density, however, higher CNT density leads to significant larger device variations over 30%, and finally resulting in lower circuits yield. Thus, we choose to use lower CNT densities for better uniformity. To test the flexibility of our CNT TFTs, the peeled-off devices were attached to a flexible polymer substrate (50 µm thickness) as the thin layer was too thin to support itself and bended to 5 mm radius (Fig. 2h). Only slight current changes were observed after many times repeated bending, suggesting the good flexibility of our devices.

### Pseudo-CMOS digital and analog circuits

**Fig. 3:**
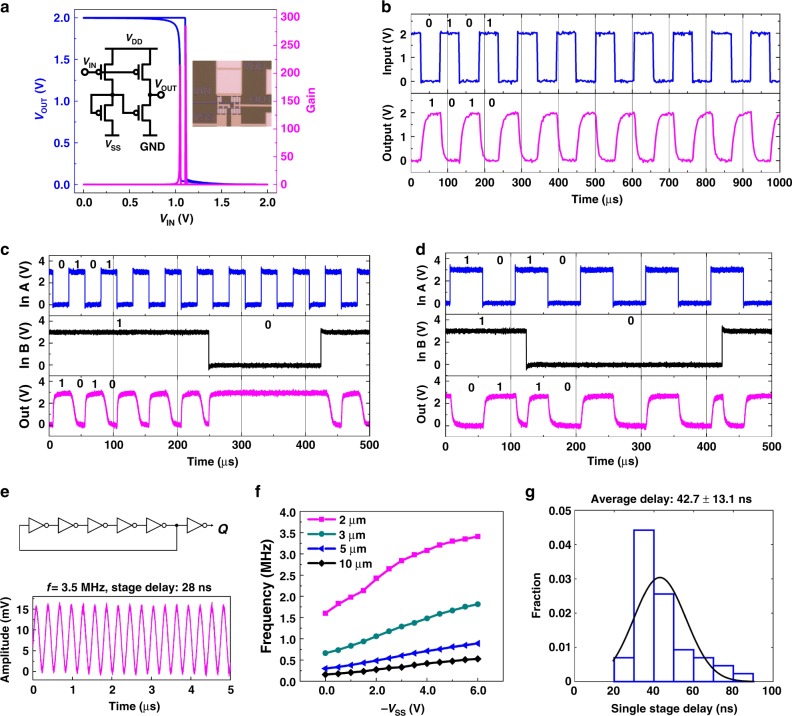
Flexible pseudo-CMOS combinational logic gates and ring oscillators. **a** Circuit diagram of using depletion-mode TFTs, die photo, input–output characteristics and small signal gain of a pseudo-CMOS inverter. Channel length (*L*) = 10 μm, *V*_DD_ = 2 V, *V*_SS_ = −3 V. **b** Measured waveforms of a pseudo-CMOS inverter running at 10 kHz. *L* = 10 μm, *V*_DD_ = 2 V, *V*_SS_ = −3 V. **c** Measured waveforms of a pseudo-CMOS NAND gate running at 20 kHz. *L* = 10 μm, *V*_DD_ = 3 V, *V*_SS_ = −3 V. **d** Measured waveforms of a pseudo-CMOS XOR gate running at 10 kHz. *L* = 10 μm, *V*_DD_ = 3 V, *V*_SS_ = −3 V. **e** Circuit diagram and output characteristics of a five-stage ring oscillator. The ring oscillator shows an oscillation frequency of 3.5 MHz with a stage delay of 28 ns. *L* = 2 μm, *V*_DD_ = 3 V, *V*_SS_ = −3 V. **f** Frequency responses of the five-stage ring oscillators under various *V*_SS_ voltages and with different channel lengths. *V*_DD_ = 3 V. **g** Stage delay statistics of a total of 44 five-stage ring oscillators with *V*_DD_ = 3 V, *V*_SS_ = −3 V. The five-stage ring oscillators show average stage delays of 42.7 ± 13.1 ns, comparable to current state-of-the-art CMOS-type CNT circuits^7^. *Note*: Results of 2 µm ring oscillators in **e** and **f** are from different devices and they are slightly different in performance due to process variations as shown in **g**. For measurement data in **e**, due to a large loading effect (measured to be ~125 pF), the measured waveform only has ~10 mV scale, which is also consistent with our simulation results in Supplementary Fig. 15

**Fig. 4:**
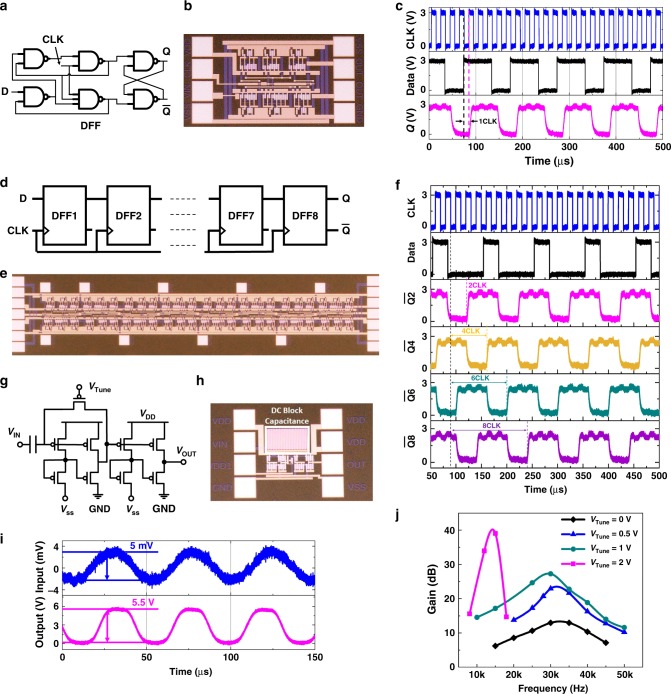
Flexible pseudo-CMOS sequential circuits and self-biased tunable gain amplifier. **a**, **b** Circuit diagram and die photo of a positive edge triggered D flip-flop (DFF). **c** Measured waveforms of a DFF with clock rate of 50 kHz and data rate of 10 kHz. *L* = 10 µm, *V*_DD_ = 3 V, *V*_SS_ = −3 V. **d**, **e** Circuit diagram and die photo of an eight-stage shift register consisting of 304 CNT TFTs. **f** Measured waveforms of an eight-stage shift register with CLK running at 50 kHz and input data running at 10 kHz. *L* = 10 µm, *V*_DD_ = 3 V, *V*_SS_ = −3 V. **g**, **h** Circuit diagram and die photo of a self-biased tunable amplifier. **i** Measured waveforms of a tunable-gain pseudo-CMOS amplifier with 5 mV input and ~5.5 V output at ~20 kHz and *V*_TUNE_ = 3 V indicating a high gain > 1000 (60 dB). *L* = 10 µm, *V*_DD_ = 6 V, *V*_SS_ = −6 V. **j** Frequency responses of a tunable amplifier with *V*_TUNE_ from 0 to 2 V. Detailed analysis of the fabricated amplifier are provided in Supplementary Fig. 17

In addition to low electronic purity of semiconducting CNTs, another obstacle to realizing a large-scale CNT TFT circuit is the lack of air-stable and high-performance n-type CNT TFTs. The instability of the n-dopant, low-work-function metals and increased complexity of the fabrication steps make the fabrication of n-type CNT TFTs much more challenging than their p-type counterparts^19,20^, leading to low circuit yields of complementary CNT circuits. To address this challenge, we employ the pseudo-CMOS design style^25^ which uses only mono-type of semiconductors, either n-type or p-type, while can achieve performance comparable to complementary-type logic circuits^25,32,33^. This alternative logic style does not only alleviate the reliance on n-type CNT TFTs, but also significantly improves the circuit speed, as well as the noise margin particularly under low supply voltages^25^. Furthermore, high uniformity and stability of our CNT TFTs enable us to build an accurate device model for circuit simulation and optimization for designing large-scale high-performance pseudo-CMOS circuits using 2-µm p-type CNT TFTs (Supplementary Note 2 and Supplementary Figs. 12 and 13). Based on these results, we show here the successful design and demonstration of several large-scale CNT-based pseudo-CMOS circuits, both analog and digital, on 4-inch flexible substrates, which include both combinational and sequential digital circuits as well as high-gain tunable amplifiers (Figs. 3 and 4).

We first built basic logic gates, including inverters, NAND, and XOR logic gates, based on which any combinational circuit can be implemented. Twelve pseudo-CMOS inverters were tested whose voltage transfer characteristics showed insignificant (0.2 V) hysteresis and the inverter’s post-fabrication tunability through adjustment of the *V*_SS_ voltage (Supplementary Fig. 14). Figure 3a shows an example of the transfer characteristics of a pseudo-CMOS inverter whose small signal gain is close to 300. The static noise margin is > 0.9 V (>90% of 1/2*V*_DD_), which is a key merit of pseudo-CMOS design for enabling large-scale CNT-based flexible circuits. We further investigated the transient behaviors of the fabricated pseudo-CMOS inverter, NAND and XOR gates, as shown in Fig. 3b–d, respectively. The measured waveforms show correct Boolean functions and rail-to-rail characteristics with 10–20 kHz inputs. Here, voltages *V*_DD_ = 3 V and *V*_GND_ = 0 V represent logic ‘1’ and ‘0’, respectively.

As shown in Fig. 3e, five-stage pseudo-CMOS ring oscillators with one output buffer stage were also designed, simulated, and fabricated. The five-stage ring oscillator showed an oscillation frequency of 3.5 MHz with a stage delay of 28 ns. We also evaluated tuning of the oscillation frequency based on the *V*_SS_ voltage as shown in Fig. 3f. As *V*_SS_ was varied from 0 to 6 V with fixed *V*_DD_ = 3 V, there exists a quasi-linear relationship between the *V*_SS_ voltage and the oscillation frequency, which is valid for different TFT channel lengths. These results indicate that the pseudo-CMOS ring oscillators can be used as voltage-controlled oscillators (VCOs) for various sensing, clocking, or communication applications. Furthermore, measured timing data for a total of 44 fabricated five-stage ring oscillators show an average stage delay of 42.7 ± 13.1 ns and the statistics is summarized in Fig. 3g. The 1056 CNT TFTs (with a 2-µm channel length) in these 44 five-stage ring oscillators fabricated on the same plastic substrate were all operating correctly, implying a >99.9% transistor yield. Compared with the recent publication^7^, the performance achieved is among the best for CNT-based flexible circuits especially for low supply voltages (Supplementary Fig. 16). We believe the achievement of such a high yield and the state-of-the-art performance is due to the combined effects of high CNT purity, TFT uniformity, and robust pseudo-CMOS design.

**Fig. 5:**
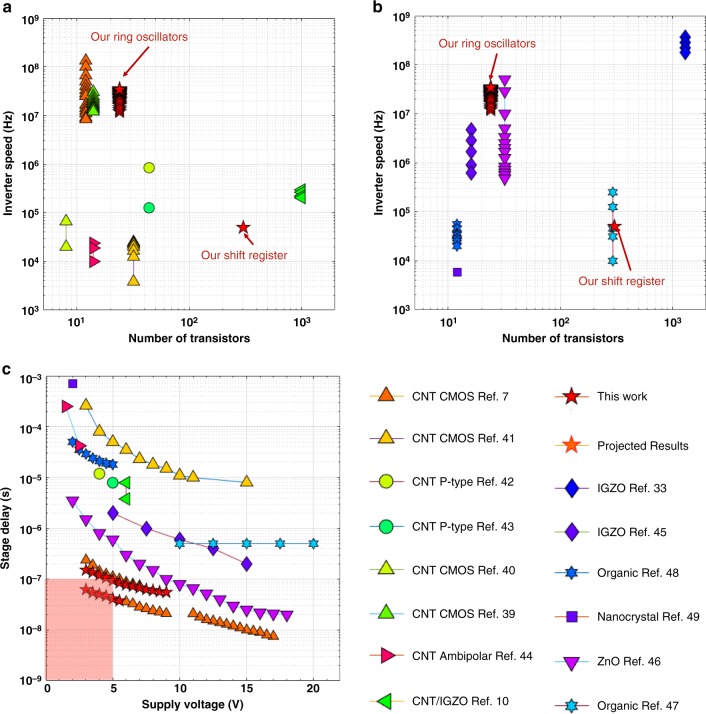
Comparison of the circuit performance and complexity of various flexible TFT circuits. **a** Comparison of mono-type, complementary, ambipolar, and hybrid CNT-based flexible circuits^7,10,39–44^. The inverter speed is defined as 1/(stage delay) and the number of transistors represents the achieved circuit complexity. **b** Comparison of state-of-the-art metal oxide, organic and nanocrystal-based flexible circuits^33,45–49^. **c** Comparison of stage delay among representative flexible ring oscillators^7,10,40–49^. To enable fair comparisons, we compared our flexible circuits with recent published results on flexible substrates not including those nanometer scale devices on rigid substrates. See Supplementary Table 3 for our CNT circuits’ typical dimensions

For CNT-based flexible circuits, while basic logic gates and sensors have been demonstrated previously^5–7,11,12^, sensor interface circuits, such as shift registers (SR), scan drivers, multiplexers, and high-gain amplifiers, were still missing. Figure 4a, b shows the circuit schematic and the die photo of a positive edge triggered D flip-flop (DFF), the key building block of SRs. The measured waveforms of the DFF is presented in Fig. 4c, where the output *Q* samples the logic value of the input data at the rising edge of the CLK and holds the data until the next rising edge. An eight-stage serial-in, parallel-out SR based on such DFFs, as shown in Fig. 4d, e, consists of a total of 304 CNT TFTs with a logic depth of 32. Figure 4f shows the measured waveforms of the SR at 50 kHz clock rate and 10 kHz data rate. The challenges of realizing fast SR at 3 V are sufficient noise, as well as setup/hold time margins when operating at fast clock rates, which set the foundation of using pseudo-CMOS CNT TFT circuits toward high-speed low-voltage flexible electronics in wearable or IoT applications.

Figure 4g, h demonstrate a CNT TFT-based voltage tunable-gain amplifier. In addition to fast and medium-complexity digital circuits, we also show the potentials of using CNT TFT for high-gain compact-sized amplifiers. We first developed an accurate CNT TFT device model, which was used for simulation and optimization of the design of a tunable-gain pseudo-CMOS amplifier. The final fabricated amplifier achieved a 1000 or 60 dB voltage gain at 20 kHz as shown in Fig. 4i, with only 9 CNT TFTs and 1 flexible capacitor. To the best of our knowledge, the measured voltage gain at given frequency ranges of this pseudo-CMOS amplifier outperforms all other flexible TFT amplifiers reported to date^13,32–38^. Also, this amplifier allows the users to fine-tune the signal gain and bandwidth by adjusting the *V*_TUNE_ node (Fig. 4g and Supplementary Fig. 17). The frequency responses of the amplifier corresponding to different voltage levels of *V*_TUNE_ are summarized in Fig. 4j. The low-frequency attenuation is due to the DC-blocking input capacitor, as shown in Fig. 4g, to avoid any DC current flowing into the input electrode and to isolate the DC bias from external sensors. In addition to being the first demonstration of using a single voltage *V*_TUNE_ to provide post-fabrication tunability for the gain and the bandwidth, the footprint of this pseudo-CMOS amplifier is also ultra-compact (350-µm^2^ excluding pads). These characteristics make this amplifier ideal for integration with various flexible sensors as a pre-amplifier to increase the signal-to-noise ratio (SNR) as well as overall sensor system sensitivity. To the best of our knowledge, this is the first demonstration of CNT-based key building blocks for sensor and display interfaces, such as SRs and high gain amplifiers^14^.

## Discussion

To illustrate the performance and the scalability advantages of our CNT TFTs and circuit solutions, we first compared our results with recent reported CNT TFT-based flexible circuits and other TFT technologies including metal oxide, organic, and nanocrystal TFTs in Fig. 5a^7,10,39–44^ and b^33,45–49^, respectively. Figure 5a clearly shows that, in terms of circuit performance and design complexity, our measured results are among the top of recent CNT TFT circuits. Although more mature metal oxide TFT technologies (blue diamond shape in Fig. 5b) show superior results, our CNT semiconducting layer is solution-processed at low temperature, which can be readily used for other scalable and low-cost fabrication technologies, such as inkjet printing and roll-to-roll fabrication^8^. The good mechanical flexibility and stretchability of CNT semiconducting networks also allow us to fabricate stretchable electronics that cannot be easily achieved using other traditional semiconductors^6,50^. Besides the demonstrated scalability and performance, our CNT-based ring oscillators firstly achieved high performance (stage delay < 100 ns) and low supply voltages (<5 V) at the same time, as indicated in the pink region of Fig. 5c, which enables the sharing of the same supply voltage and easier integrations with silicon chips (no need for power conversions). In light of the high complexity and low device yield of the integration of n-type CNT TFTs, our pseudo-CMOS design provides an alternative device-to-circuit solution to realize comparable or even higher circuit speed than complementary design, though several limitations still exist, including higher power consumption, larger circuit area, and additional wiring.

In conclusion, we have developed a novel sorting method that can achieve high purity (99.997%) and high yield (19.9%) separation of semiconducting CNTs. Systematic device fabrication and optimization for CNT deposition, dielectric/semiconductor interface, device configuration, and encapsulations lead to high device yield, uniformity, and much better bias stability. Using the developed transistor model and pseudo-CMOS design style, we have demonstrated the design, optimization, and fabrication of CNT-based circuits with superior performance and scale operating at low supply voltages. These advances can further enable new innovations in flexible CNT-based sensor acquisition systems.

## Methods

### Materials

Polymer PFPD was synthesized according to our reported method^26^. 11-(2-methoxyethoxy) undecyltrimethoxysilane (CAS number: 1384163-86-3) was purchased from Gelest Inc. and used as received. mr-DWL_1 XP photoresist was purchased from Micro Resist Technology GmbH.

### Improved CNT sorting method using degradable conjugated polymers

5 mg of PFPD and 15 mg of crude SWNTs (RN-020, purchased from Raymor Industries) were mixed in 25 mL of toluene and ultrasonicated for 30 min at an amplitude level of 50% (Cole Parmer ultrasonicator 750 W) while externally cooled with a dry ice bath. The mixture was centrifuged at 8000 rpm for 5 min to remove most of the undispersed residues, and then centrifuged at 17,000 rpm (22,000 × *g*) for 25 min at 16 °C, 90% of the supernatants (~22 mL) was collected. The supernatants were stored in a fridge (4 °C) for 24 h to allow the metallic tubes to further aggregate. After mildly sonicating the supernatants in a bath sonicator for 5 min, the solution was centrifuged at 17,000 rpm again for 30 min at 16 °C. Finally, 90% of the supernatants (~20 mL) were collected, which are ready for CNT device fabrication.

### CNT purity characterization

Since the CNT purity is already very high (>99%) and optical characterization methods (e.g. absorption and Raman spectroscopy) cannot provide accurate estimation of the CNT purities, direct electrical measurement of single tubes using short channels are used. First, diluted SWNT solutions (20 times) were drop-casted on thermally grown SiO_2_ (36 nm)/Si wafer, waited for 20 s, then gradually ramp spin speed to 3000 rpm until the surface is dry. Removal of wrapping polymers from SWNTs was carried out by rinsing with toluene containing a small amount (0.1% v/v) of TFA and rapid thermal annealing (RTA) of wafer for 15 min in N_2_ at 500 °C. Palladium (30 nm) as metal contact was defined by traditional photolithography process using an ASML 5500 Stepper. Using a chuck to globally bias the wafer as a gate, the electrical behavior of the CNTs was measured using an Agilent B1500 and an automatic probe station. Since most of the sorted tubes exhibited tube lengths in the range of 0.5–2 μm, the short channel devices used here will short if there are m-SWNTs bridging directly across the channels.

Two batches of short-channel (500 nm) transistors were fabricated, one had 1049 FETs (batch #1, density: 4.5 CNTs/FET), and another had 7998 FETs (batch #2, density: 5.8 CNTs/FET). Among these transistors, some showed low on-current (<0.1 μA) due to percolation transport rather than direct CNT connection. Therefore, only 1023 transistors in batch #1 and 5170 transistors in batch #2 were used for evaluation. The number of CNTs used for evaluation is calculated as [1023 × 4.5 + 5170 × 5.8 = 34,590]. Among the 34,590 tested CNTs, only 1 metallic tube was found in batch #2. The purity is thus estimated to be 99.997%.

### Fabrication of flexible CNT circuits on polyimide substrate

To a clean 4-inch wafer, 5 mL PI-2611 (polyimide, HD MicroSystems) was dispensed and spin-coated at 2000 rpm for 30 s. The device was baked at 90 °C for 2 min and 150 °C for 3 min. The wafer was then placed in oven with nitrogen ambient. The oven temperature ramped up from room temperature to 350 ^°^C at 2 °C per minute and was held for 30 min to fully cross-link the polyimide layer. 50-nm SiN_*x*_ layer was deposited as the barrier layer using plasma-enhanced chemical vapor deposition (PECVD). The metal_layer_1 was patterned by photolithography and 35-nm Cr layer was deposited using e-beam evaporation, followed by lift-off process. A conformal Al_2_O_3_ (20 nm) and SiO_2_ (5 nm) stack was deposited by ALD as the gate dielectric over the substrate at 200 °C. Gate contact vertical interconnect accesses (VIAs) were patterned by photolithography and wet etching using 20:1 BOE etchant for 10 s and standard aluminum etchant for 5 min. Photolithography, e-beam evaporation (2 nm Ti/40 nm Pd) and lift-off in acetone were used to define the metal_layer_2. The device was modified with the SAM layer by spin-coating the silane solution (3 mM in trichloroethylene) at 2000 rpm and baked at 120 °C in air for 10 min. The polymer sorted SWNTs were deposited by soaking the wafer in the 40 mL SWNT (~0.02 mg/mL) solution for 30 min, followed by rinsing with toluene (2 min), 0.1% v/v TFA in toluene (2 min), and then toluene (2 min) to remove the wrapping polymers. After SWNT deposition, the substrate was annealed at 200 °C for 5 min. This process was repeated once to enhance the CNT uniformity and density. A negative photo resist mr-DWL_1 XP was coated with 1 μm thickness and patterned by photolithography. Oxygen plasma was then used to pattern the CNT layer. To fully encapsulate the device, 40 nm ALD Al_2_O_3_ was deposited at 100 ^°^C. This layer was then patterned using photolithography and wet etching with aluminum etchant to give VIAs for measurement. The polyimide layer can be pealed-off to give the final flexible CNT circuits.

### Device testing

The transfer, leakage, and bias stability characteristics of the CNT TFTs and static voltage transfer curves of pseudo-CMOS inverters were measured using Agilent 4156C Semiconductor Analyzer. Transient waveforms of logic gates, SRs and amplifiers were measured with Agilent 4156C Semiconductor Analyzer, Agilent 33250A Waveform Generator and LeCroy 104MXi-A Oscilloscope. The DC supplies and transient stimulus were provided by Agilent 4156C and Agilent 33250A, respectively, and waveforms were captured by LeCroy 104MXi-A.

## Supplementary information

Supplementary Information

## Data Availability

The authors declare that the main data supporting the findings of this study are available within the article and its Supplementary Information files. Extra data are available from the corresponding author upon request.
