# Supplementary material for: Low-voltage high-performance flexible digital and analog circuits based on ultrahigh-purity semiconducting carbon nanotubes
Source: Nat Commun. 2019 May 14;10:2161. doi: 10.1038/s41467-019-10145-9 (PMC6517392; doi:10.1038/s41467-019-10145-9)
Supplement: Supplementary file 1 — Supplementary Information [file 41467_2019_10145_MOESM1_ESM.pdf]

Supplementary Information for

Low-voltage High-performance Flexible Digital and Analog Circuits based on Ultrahigh-purity Semiconducting Carbon Nanotubes

Ting Lei[1,2†], Lei-Lai Shao[3,5†], Yu-Qing Zheng[1], Gregory Pitner[4], Guanhua Fang[6], Chenxin Zhu[1], Sicheng Li[5], Ray Beausoleil[5], Hon-Sum Philip Wong[4], Tsung-Ching Huang[5,], Kwang-Ting Cheng[3,7,*] and Zhenan Bao[1,*]*

[1]Department of Chemical Engineering, Stanford University, Stanford, CA 94305, USA

[2]Department of Materials Science and Engineering, College of Engineering, Peking University, Beijing 100871, China

[3]Department of Electrical and Computer Engineering, University of California, Santa Barbara, CA 93106, USA

[4]Department of Electrical Engineering, Stanford University, Stanford, CA 94305, USA

[5]Hewlett Packard Labs, Palo Alto, CA 94304, USA

[6]Department of Materials Science & Engineering, Stanford University, Stanford, CA 94305, USA

[7]School of Engineering, Hong Kong University of Science and Technology, Hong Kong, China

[†]These authors are contributed equally to this work.

This file includes:

1. Supplementary Figures
2. Supplementary Tables.
3. Supplementary Notes
4. Supplementary References

1. Supplementary Figures:

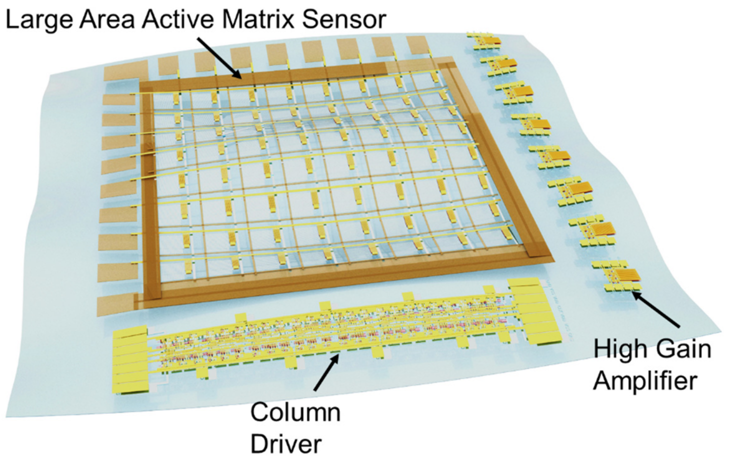

Supplementary Figure 1| A conceptual flexible sensor acquisition system. A 2-D flexible sensor array integrated with flexible column drivers and near sensor flexible amplifiers.

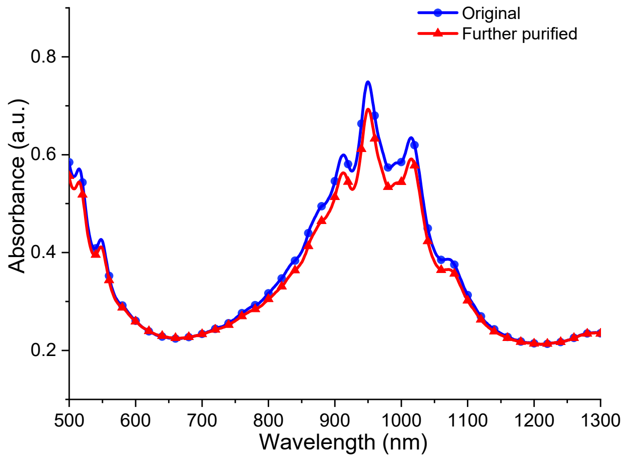

Supplementary Figure 2| Absorption spectrum comparison of the original sorted solution and further purified solution. We estimate the solution concentration using the maximum peak intensity. The CNT concentration of the further purified solution is about 84% of the original one, suggesting that ~16% of the s-SWNTs were lost during the further purification.

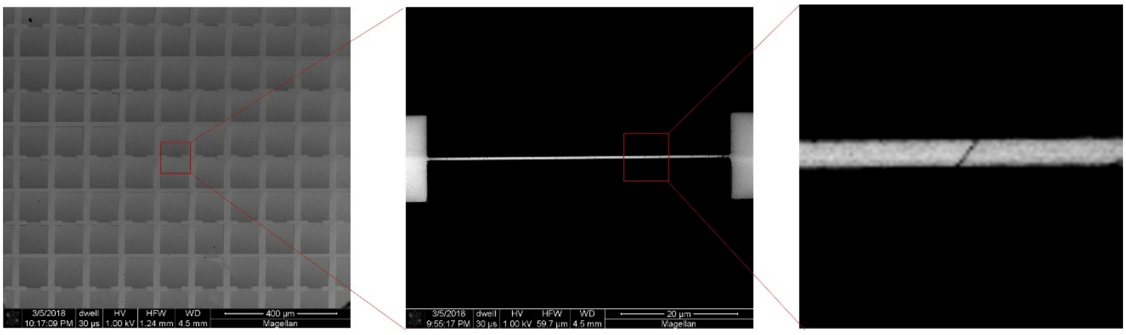

Supplementary Figure 3| SEM images of the short channel device matrix used for CNT purity evaluation. CNT can be clearly seen from the SEMs and the number of CNTs can be counted, and the average CNTs per device can be estimated.

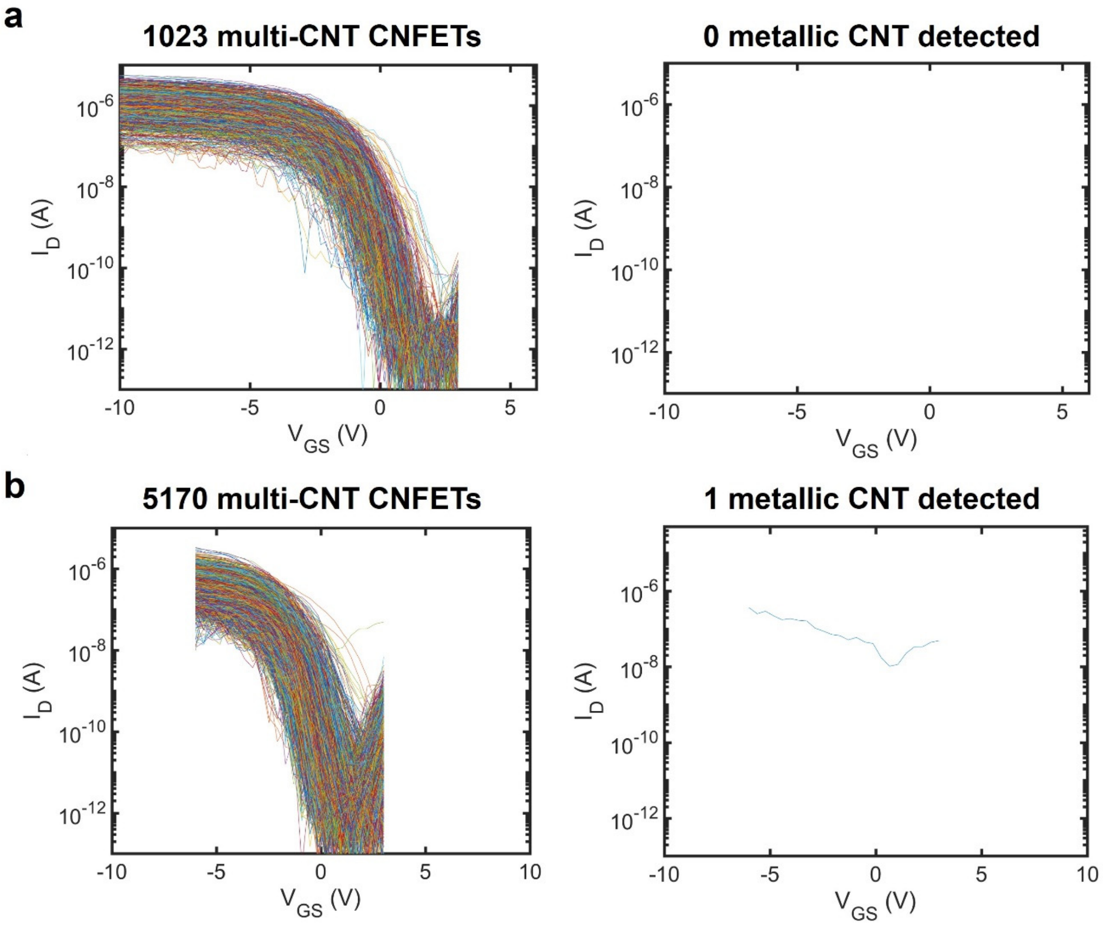

Supplementary Figure 4| CNT purity evaluation using electrical measurement method. a, 1023 transistors in batch #1 and **b**, 5170 transistors in batch #2 were measured

and used for evaluation. The number of CNTs used for evaluation is calculated as [1023 × 4.5+5170 × 5.8 = 34590]. Among the 34590 tested CNTs, only 1 metallic tube was found in batch #2. The purity is thus estimated to be 99.997%.

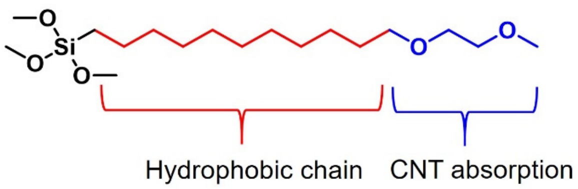

Supplementary Figure 5| Chemical structure of 11-(2-methoxyethoxy) undecyltrimethoxysilane used for the SAM layer modification. This molecule has both hydrophobic and hydrophilic parts. The hydrophobic part is used to reduce the hysteresis, while the hydrophilic part is used to enhance the CNT absorption.

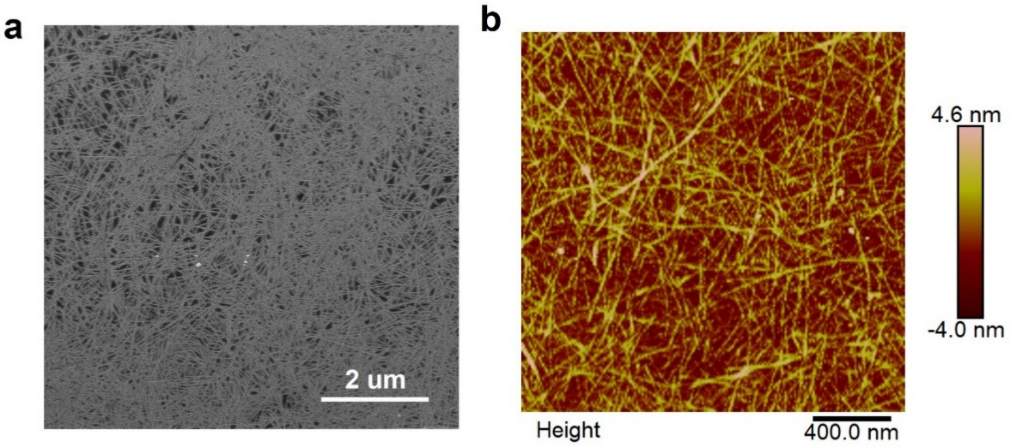

Supplementary Figure 6| a, SEM and **b**, AFM images of the deposited CNT thin films. The CNT line density is estimated to be 35~40 CNTs/µm.

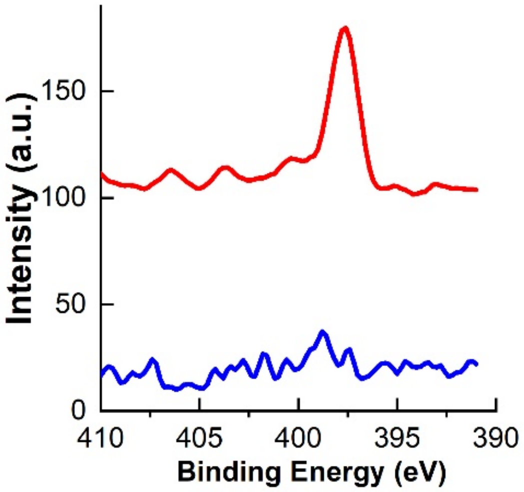

Supplementary Figure 7| X-ray photoelectron spectra for the deposited CNTs wrapped with PFPD polymers (red) and the CNTs after rinsing with 0.1 % v/v trifluoroacetic acid (TFA) in toluene (blue). The nitrogen 1s peak (397.6 eV) on the polymer disappeared after polymer removal, indicating that the wrapping polymers were mostly removed after acid treatment.

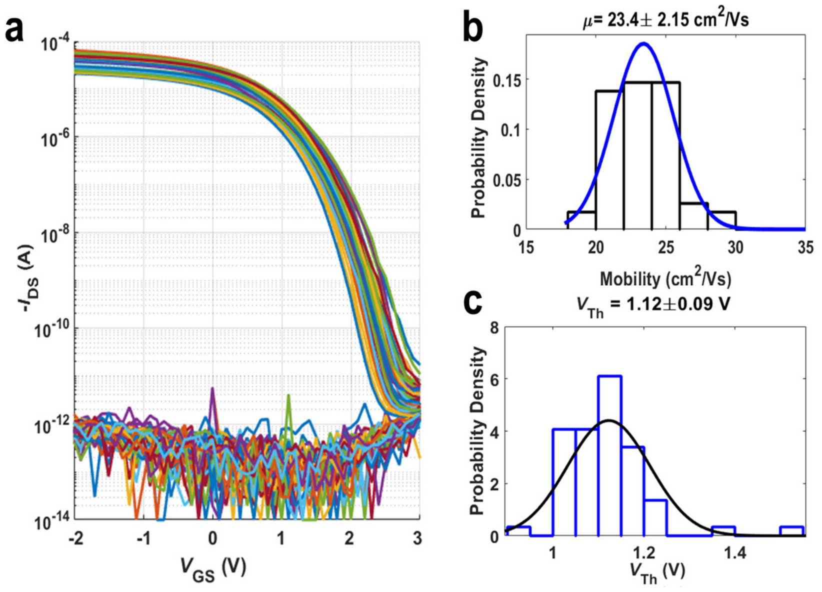

Supplementary Figure 8| Typical 10um CNT TFTs' transfer characteristics on a 4-inch wafer with V_{DS}=-0.5 V. a, Transfer characteristics of CNT TFTs with W=50 um. 56

transistors show on-off ratios >10^6 and gate leakages ~ 1pA. **b&c,** Extracted mobility and threshold voltage (V_{Th}) based on traditional CMOS linear region model.

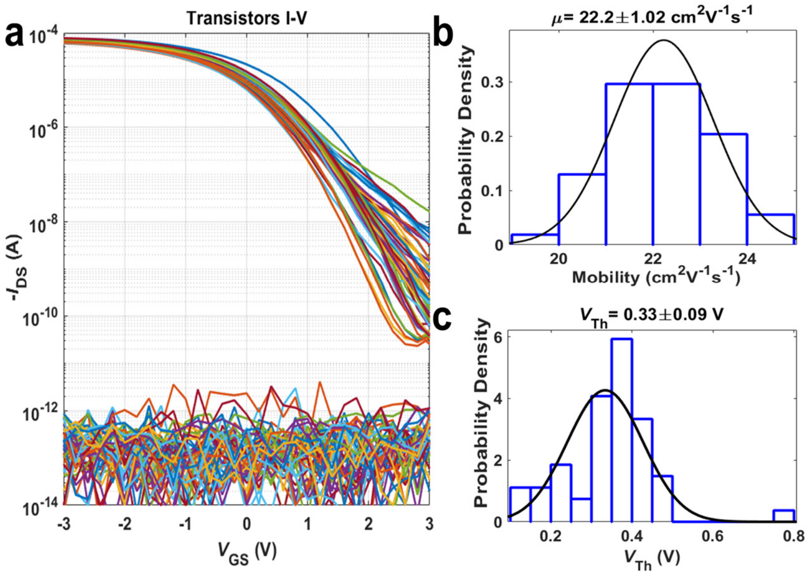

Supplementary Figure 9| Typical 2um CNT TFTs' transfer characteristics with V_{DS}=-0.5 V. a, Transfer characteristics of CNT TFTs with *W*=25 um. 54 transistors show on-off ratios >10^5 and gate leakages ~ 1pA. **b&c,** Extracted mobility and threshold voltage (V_{Th}) based on traditional CMOS linear region model.

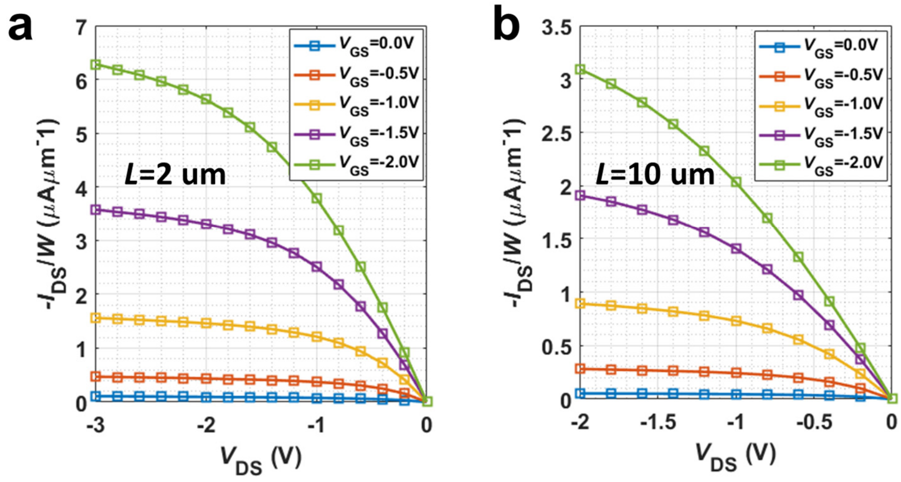

Supplementary Figure 10| Current density of typical CNT transistor with *L* = 2 um and *L* = 10 um.

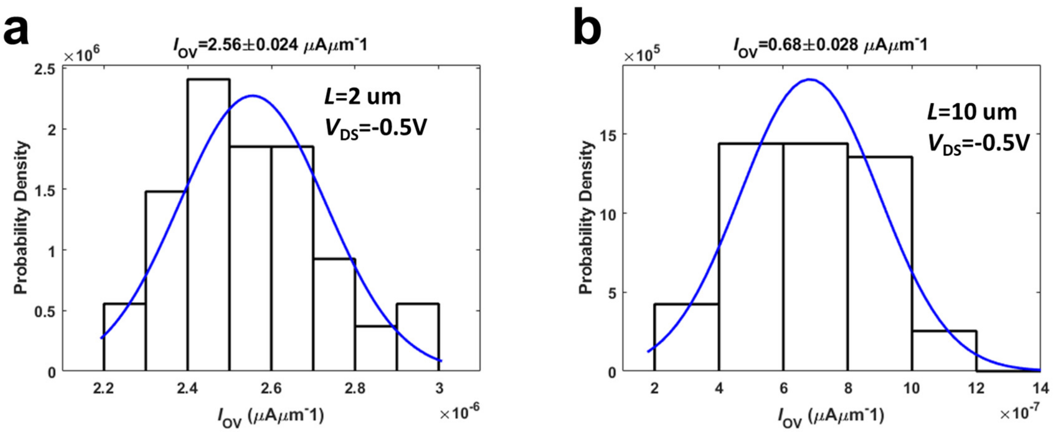

Supplementary Figure 11| Overdrive current density (|V_{GS}-V_{Th}|=1V) of typical CNT transistor with *L*=2um and *L*=10um.

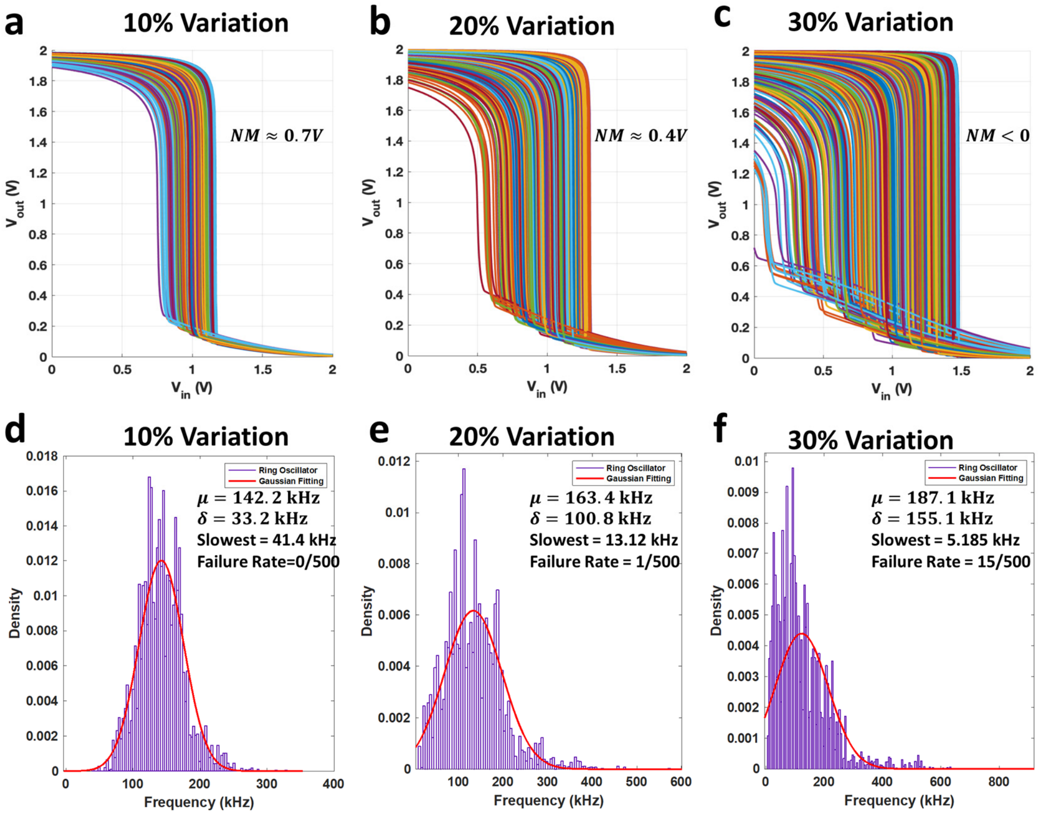

Supplementary Figure 12| Monte Carlo simulations for noise margin (NM), circuit speed and circuit yield under different process variations: 10%, 20% and 30%. a-c, 500 Monte Carlo simulations are performed to analyze the NM of Pseudo-CMOS inverters.

Noise margin of the inverter drops dramatically from 70%(1/2 V_{DD}) to <0% as the process variation increases from 10% to 30%, which indicates the importance of variation control for large NM/robust circuit design. On the other hand, our high uniform (<10% variation) CNT-TFTs can give us a high NM, which enables our designed circuit to operate at a low supply voltage (<3 V). **d-f,** Circuit speed and yield analysis for 5-stage ring oscillators. Similarly, 500 Monte Carlo simulations are conducted for 5-stage ring oscillators for speed and yield analysis.

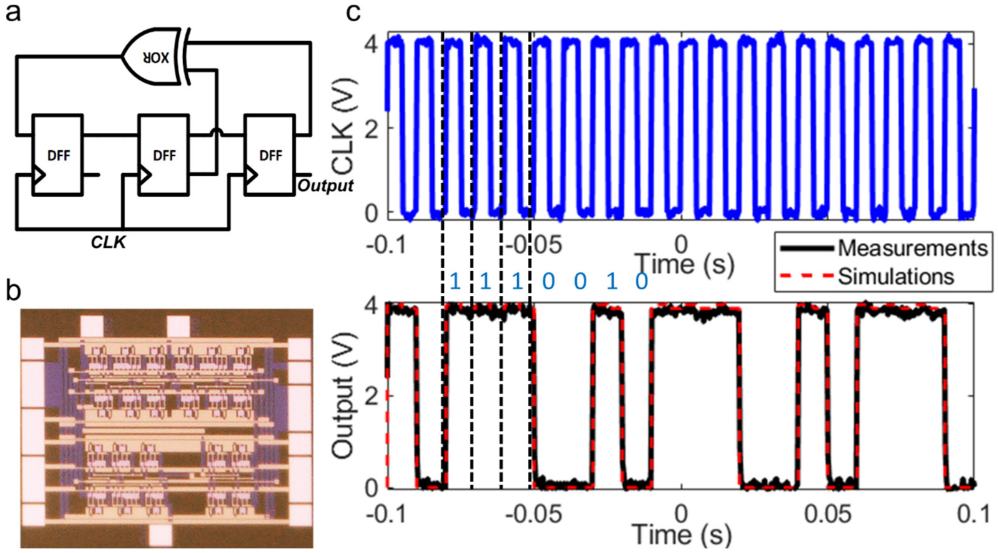

Supplementary Figure 13| Simulations and measurements of a 3-bit maximum-length linear feedback shift register (LFSR). LFSR based random sequence generator, which can be applied in signature generation, cryptography and test-patter generation. **a&b,** Schematic and die photo of a 3-bit LFSR sequence generator. **c,** Measured and simulated waveforms of a generated 7-bit sequence ('1110010') with *CLK*=100 Hz, V_{DD}=4 V and V_{SS}=-4 V.

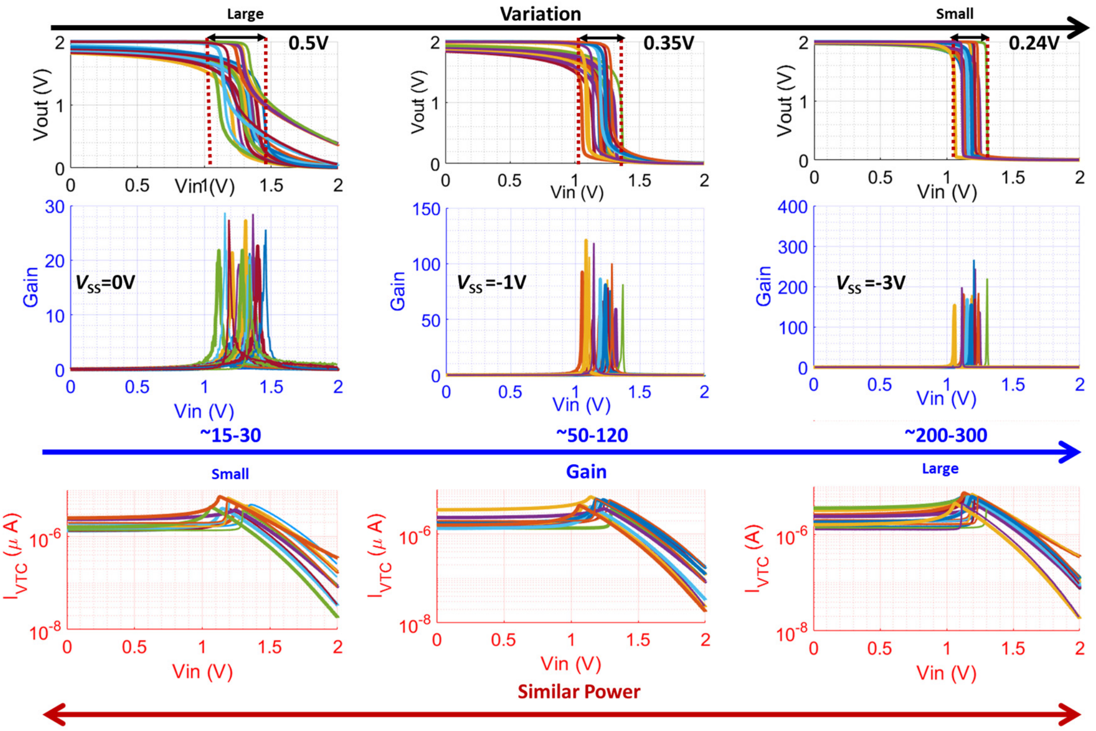

Supplementary Figure 14| Post-tuning of 12 pseudo-CMOS inverters by varying V_{SS}. Pseudo-CMOS inverters' transfer characteristics with various V_{SS} and fixed V_{DD}=2 V. By changing V_{SS} from 0 V to -3 V, transfer curves' variations are reduced from ~0.5V to ~0.2V and small signal gains boost from ~15-30 to ~150-300. Also, only slightly increases of the static currents are observed under different V_{SS}.

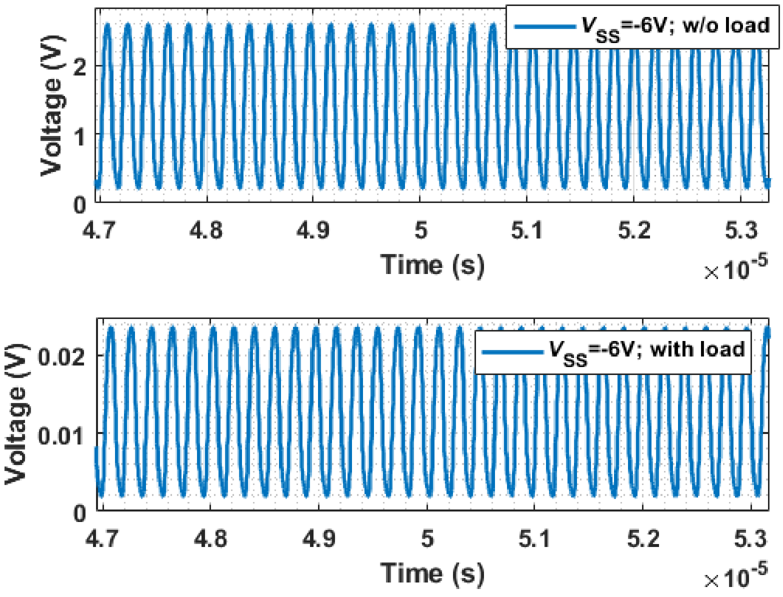

Supplementary Figure 15| Simulated dynamic waveforms of a ring oscillator with or w/o loads. For measurement data, due to a large loading effect (measured to be ~125 pF), the measured waveform only has ~10 mV scale, which is also consistent with our simulation results.

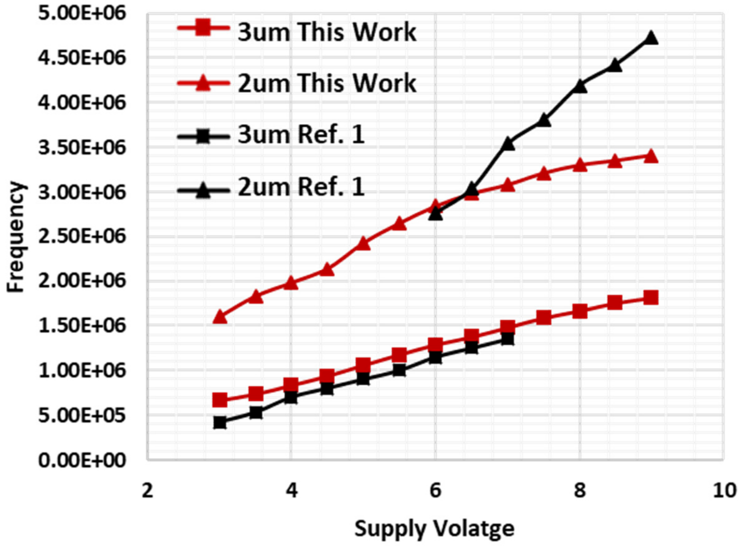

Supplementary Figure 16| Circuit performance comparison to current state-of-art results of flexible CNT TFTs[1]. In this figure, we compare our 5-stage pseudo-CMOS ring oscillator results to Ref. 1 using CMOS design. We achieved comparable or even better

results. Especially for low supply voltage (3-5V)[a], our 2-um ring oscillators achieved record-high performance, as fast as 2.4 MHz, while Ref. 1 fails to report competitive results due to thick dielectric layers. Note that low supply voltages[2,3] (sub-5 V) is crucial to enable integrations with thin film flexible battery and silicon ICs without complicate power conversion overheads. In addition, low supply voltages are also safer for wearable and implant applications.

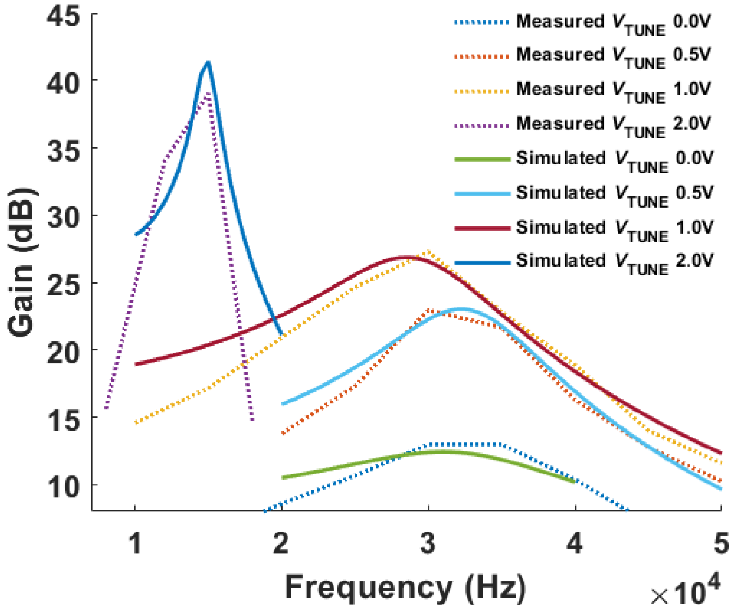

Supplementary Figure 17| Measured and simulated frequency responses of a tunable gain amplifier. The self-biased tunable amplifier is composed of two stages. The first stage is based on a Pseudo-CMOS inverter with a shunt-shunt feedback CNT transistor biased in the linear region, as well as an input capacitor to block the DC current flowing from the inverter output to the input terminal V_{IN}. The input capacitor also attenuates the low-frequency input signals and the size of the capacitor determines the dominant zero location in the frequency responses, which explains the rising gain response from 10KHz to 30KHz. The feedback linear CNT transistor serves two purposes: biasing the Pseudo-CMOS inverter to be in the high-gain region around half-V_{DD}, and also providing a resistive feedback to form a feedback amplifier. Similar to typical amplifier with a shunt-shunt

[a] To enable fair comparisons, we use $|V_{DD}-V_{SS}|$ as our supply voltage for pseudo-CMOS circuits.

feedback resistor, the closed-loop gain of the first stage will be approximately the resistivity of the feedback linear CNT transistor. While the resistivity of the linear feedback transistor depends on V_{TUNE}, the closed-loop gain of the first stage amplifier with the linear feedback CNT transistor will also vary according to V_{TUNE} voltages. On the other hand, the bandwidth of such feedback amplifier also depends on the resistive feedback. The pole locations in the frequency response will move to lower frequencies as the resistivity of the feedback linear CNT transistor increases, which explains the smaller bandwidth with higher V_{TUNE} voltages. Although in theory the relationship between V_{TUNE} voltages, the resistivity of the feedback CNT transistor biased in the linear region, as well as the closed-loop midland gain should be linear, the bandwidth of such amplifier also changes with V_{TUNE} voltages. Therefore, the proposed self-biased amplifier based on Pseudo-CMOS inverter is better suited for narrowband signals, while it can be easily modified for wideband amplification after removing the input capacitor. The second stage of the self-biased amplifier is simply a Pseudo-CMOS inverter, which is biased around half-V_{DD} at the high-gain region. The second stage will therefore work as common-source amplifiers with fixed voltage gains. After correlating our developed SPICE model with CNT TFT's measurements, the simulation results can predict the basic trend of the fabricated amplifier. While increasing V_{TUNE} ($|V_{GS}|\downarrow$), the effective resistance of the feedback CNT TFT decreases. According to the simulation results, when V_{TUNE}=2V, the feedback TFT enters the near-subthreshold region leading to a dramatic change in the effective resistance, which cause a jump in the frequency response.

2. Supplementary Tables:

Supplementary Table 1| CNT TFT comparisons for mobility, variation and on-off ratio.

References	Mobility (cm^2/Vs)	Variation[b] (σ/μ)	On-off Ratio	L (um)
Ref 4	50(p type)	~15%	10^4-10^2	100 to 4
Ref 5	35(p type)	NA	10^6-10^1	100 to 5
Ref 1	40(n type) / 55(p type)	~20%	10^6-10^4	8 to 2
Ref 6	9(p type)	~30%	10^5	85
Ref 7	9(n type) / 15(p type)	NA	10^5	20
Ref 8	9(p type)	~20%	10^5	20
Ref 9	20(n type) / 30(p type)	~25%	10^4	50
**This work**	**25(p type)**	**<10%**	10^5	**10 to 2**

Supplementary Table 2| Comparison of current density.

References	Current Density ($\mu A * \mu m^{-1}$)	On-off Ratio	L (um)
Ref 1	17	10^6	2
Ref 5	0.2	10^4-10^2	100 to 4
Ref 6	0.02	10^5	85
Ref 7	0.01	10^5	20
Ref 9	1-7	10^6-10^4	8 to 2
Ref 10	0.01-3	10^4-10^2	100 to 4
Ref 11	0.3	10^6-10^1	100 to 5
Ref 12	0.1	10^6-10^4	8 to 2
Ref 13	10	10^5	20
Ref 14	0.1	10^4	50
Ref 15	0.3	10^6-10^1	100 to 5
Ref 16	1-10	10^5	85
**This work**	**6**	$\mathbf{10^5}$	**2**

Supplementary Table 3| CNT circuits' typical dimensions[c].

[b] Due to limited publication data, we use the mobility to represent overall variations.

[c] We include the normalized transistor count, in unit of the minimum-sized transistor, for each circuit. The normalized transistor count is a more comprehensive way to represent the actual required area/power for logic gates. To optimize for the speed and noise margin, M2-M4 in a pseudo-CMOS inverter are typically composed of three 50 um parallel transistors in ours design. Thus, the normalized transistor number is 10 for each inverter.

Circuit Type	Schematic	Dimensions	Normalized Transistor Count	Rough Transistor Count	Related Figures
Inverter	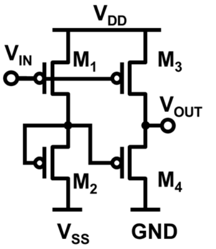 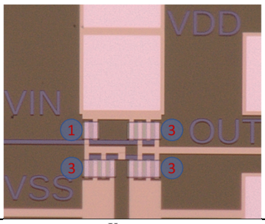	L=10um/2um<br>M1=50um<br>M2=150um<br>M3=150um<br>M4=150um	10	4	Figure 3<br>Supplementary Figure 12<br>Supplementary Figure 14<br>Supplementary Figure 16
NAND Gate	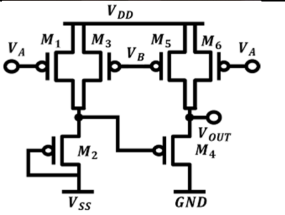	L=10um<br>M1=50um<br>M2=50um<br>M3=50um<br>M4=100um<br>M5=100um<br>M6=100um	9	6	Figure 3
XOR Gate	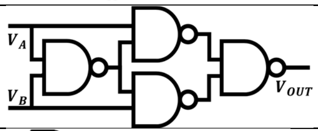	L=10um<br>Formulated by four NAND gates as above	36	24	Figure 3<br>Supplementary Figure 13
DFF	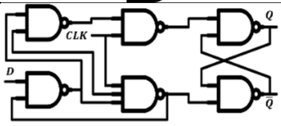	L=10um<br>Formulated by five 2-inputs NAND (9 TFTs) gates and one 3-inputs NAND (12 TFTs) gates	57	38	Figure 4<br>Supplementary Figure 13
8-Stage Shift Register	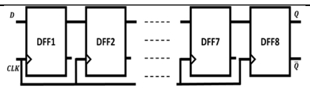	L=10um<br>Cascaded by 8 DFFs	456	304	Figure 4
Amplifier	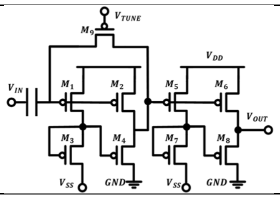	L=10um<br>M1, M5, M9=50um<br>Others=150um<br>C≈1nF	21	9	Figure 4<br>Supplementary Figure 17

3. Supplementary Notes:

Supplementary Note 1: Device yield estimation based on simulation

From Supplementary Figure 12 e-f, we can clearly see that ring oscillator's frequency becomes more widespread indicating a larger circuit level variation as the process variation increases. The lower bound of the ring oscillator determines the overall system performance since generally the slowest component becomes the bottle neck. Thus, from system performance point of view, process variation control is also a critical factor to achieve high performance and large-scale circuits. For circuit yield, some ring oscillator fails to work under 20% and 30% variation, which poses a strict requirement of process variation for large scale circuits. Our high-performance (>50 kHz) low-voltage (<3V) 8-stage shift register (containing >300 CNT-TFTs) shows strong evidence of the great uniformity of our CNT devices.

Supplementary Note 2: Transistor modeling and circuits design

To enable circuit simulations, a behavior model of CNT TFTs is developed using Verilog-A integrated with Cadence Virtuoso, a design platform for silicon integrated circuit design. The transistor model can accurately predict the outcome of CNT TFT based circuits without fabrication and allows designers to optimize the noise margin (NM) and performance by choosing appropriate parameters and transistor sizes. First, we will optimize the NM through extensive simulations of different transistor ratios. Once the ratio is fixed, we further optimize the speed or power via parameters sweeping of the transistor's channel length and width. The good match between our CNT TFTs and behavior model dramatically reduces the design efforts and challenges. Furthermore, the whole design flow is fully automatic via our developed optimization program.

As a design example, simulation results of a linear feedback shift register (LFSR) match closely with measurements, as shown in Supplementary Figure 13c. Benefiting from uniform and stable devices and the accurate transistor model, we successfully demonstrated large scale CNT TFT based circuits (Fig. 3&4). The developed model and various design examples, such as inverter, NAND, XOR, DFF and SR, will be open sourced (https://github.com/llshao/CNT-TFT-Verilog-A) to facilitate the CNT TFT based large-scale circuits design.

4. Supplementary References

1. Tang, J. *et al.* Flexible CMOS integrated circuits based on carbon nanotubes with sub-10 ns stage delays. *Nat. Electron.* **1,** 191 (2018).
2. Takei, K. *et al.* Nanowire active-matrix circuitry for low-voltage macroscale artificial skin. *Nat. Mater.* **9,** 821 (2010).
3. Ha, M. *et al.* Aerosol jet printed, low voltage, electrolyte gated carbon nanotube ring oscillators with sub-5 μs stage delays. *Nano Lett.* **13,** 954–960 (2013).
4. Wang, C. *et al.* Extremely bendable, high-performance integrated circuits using semiconducting carbon nanotube networks for digital, analog, and radio-frequency applications. *Nano Lett.* **12,** 1527–1533 (2012).
5. Sun, D. *et al.* Flexible high-performance carbon nanotube integrated circuits. *Nat. Nanotechnol.* **6,** 156 (2011).
6. Lau, P. H. *et al.* Fully printed, high performance carbon nanotube thin-film transistors on flexible substrates. *Nano Lett.* **13,** 3864–3869 (2013).
7. Zhao, Y. *et al.* Three-dimensional flexible complementary metal–oxide–semiconductor logic circuits based on two-layer stacks of single-walled carbon nanotube networks. *ACS Nano* **10,** 2193–2202 (2016).
8. Chen, H., Cao, Y., Zhang, J. & Zhou, C. Large-scale complementary macroelectronics using hybrid integration of carbon nanotubes and IGZO thin-film transistors. *Nat. Commun.* **5,** 4097 (2014).
9. Ha, M. *et al.* Printed, sub-3V digital circuits on plastic from aqueous carbon nanotube inks. *ACS Nano* **4,** 4388–4395 (2010).
10. Chandra, B., Park, H., Maarouf, A., Martyna, G. J. & Tulevski, G. S. Carbon nanotube thin film transistors on flexible substrates. *Appl. Phys. Lett.* **99**, 072110 (2011).
11. Tian, B. *et al.* Wafer scale fabrication of carbon nanotube thin film transistors with high yield. *J. Appl. Phys.* **120**, 034501 (2016).
12. Cao, Q. *et al.* Medium-scale carbon nanotube thin-film integrated circuits on flexible plastic substrates. *Nature* **454**, 495-500 (2008).
13. Honda, W., Arie, T., Akita, S. & Takei, K. Mechanically Flexible and High-Performance CMOS Logic Circuits. *Scientific Reports* **5**, 15099 (2015).
14. Wang, H. *et al.* Tuning the threshold voltage of carbon nanotube transistors by n-type molecular doping for robust and flexible complementary circuits. *Proc. Natl. Acad. Sci. USA* **111**, 4776-4781 (2014).
15. Chen, H., Cao, Y., Zhang, J. & Zhou, C. Large-scale complementary macroelectronics using hybrid integration of carbon nanotubes and IGZO thin-film transistors. *Nat. Commun.* **5**, 4097 (2014).
16. Wang, C. *et al.* Extremely Bendable, High-Performance Integrated Circuits Using Semiconducting Carbon Nanotube Networks for Digital, Analog, and Radio-Frequency Applications. *Nano Lett.* **12**, 1527-1533 (2012).
